# Mapping Nurses' Knowledge, Attitudes, and Practices to Guide Competency-Based Infection Control Training

**DOI:** 10.7759/cureus.95258

**Published:** 2025-10-23

**Authors:** Renu Gupta, Ranga Reddy Burri, Srivalli Bhagavatula, Abhilash Patra, Rupjyoti Chandok, Insha Altaf, Debolina Halder

**Affiliations:** 1 Microbiology, Institute of Human Behaviour and Allied Sciences, Delhi, IND; 2 Infection Prevention and Control, Infection Control Academy of India, Hyderabad, IND; 3 Public Health, University of Hyderabad, Hyderabad, IND; 4 Microbiology, Blue Flower Children’s Hospital and Diagnostics, Vijayawada, IND; 5 Biostatistics, Pragyaan Sustainable Health Outcome Foundations, Hyderabad, IND; 6 Microbiology, Max Super Speciality Hospital, Saket, Delhi, IND; 7 Microbiology, Paras Health, Srinagar, IND; 8 Nursing, The Mission Hospital, Durgapur, IND

**Keywords:** attitudes, competency based education, education, infection control, infection prevention, knowledge, nurse attitude, nursing curriculum, nursing staff education, practice

## Abstract

Aim/objective: To assess infection prevention and control (IPC) knowledge, attitudes, and practices (KAP) among Indian nurses and identify key competency gaps to inform standardized training.

Background: Healthcare-associated infections (HAIs) are a persistent global challenge, posing serious risks to patient safety and health system performance. Nurses, as frontline caregivers, have a critical role in IPC. In India, however, the lack of a nationally standardized IPC competency framework and the resulting disconnect between formal nursing education and real-world clinical practice lead to inconsistent implementation and preventable harm. Integrating evidence-based, context-sensitive IPC standards into nursing curricula and ongoing professional training is essential to ensure that best global practices are consistently applied and to strengthen patient safety across diverse healthcare settings.

Design and methods: The study was designed as a nationwide cross-sectional survey, involving a structured online questionnaire, validated against WHO and CDC IPC guidelines, that surveyed 547 Indian nurses across diverse hospitals and regions. Knowledge, attitude, and practice (KAP) scores were categorized as low, average, or good. Descriptive statistics, chi-square tests, and ordinal logistic regression were used for analysis.

Results and discussion: The mean KAP score was 33 ± 5.39 out of 60 (54.73%). Attitude emerged as the weakest domain (45.5%), with knowledge (57.4%) and practice (58.6%) showing average competence. While nurses were confident in standard precautions and transmission chains, many lacked awareness of device reprocessing, environmental cleaning, PPE use, and elements of antimicrobial stewardship. Practice gaps, particularly in hand hygiene and waste management, were identified and appear driven by resource constraints, high patient workloads, and insufficient institutional support. Differences in performance related to experience and workplace setting highlight the critical influence of training quality, exposure, and organizational factors.

Conclusion: This study reveals significant IPC competency gaps among Indian nurses, driven not just by knowledge deficits but by barriers to translating knowledge into daily behaviors. Closing these gaps requires standardized, practical IPC education, targeted certification, and strong institutional mechanisms. The lessons and recommendations from this study, although focused on India, are highly relevant to health systems throughout the global south facing similar challenges in infection control.

## Introduction

Healthcare-associated infections (HAIs) are among the most frequent and serious adverse events in healthcare delivery, posing a major threat to patient safety and contributing substantially to global morbidity, mortality, and healthcare costs [1,2]. On average, one in 10 patients is affected by HAI, with a much higher frequency in low/middle-income countries, underscoring its widespread impact on healthcare quality [1]. The burden is especially pronounced in high-risk settings such as intensive care units (ICUs), where patients are particularly susceptible to opportunistic pathogens due to the severity of illness and invasive interventions. Infection prevention and control (IPC), therefore, remains a clinical and public health priority with significant implications for health system performance and economic sustainability [1,2].

Nurses, being at the forefront of patient care, play a pivotal role in IPC implementation. However, in India, the lack of a nationally defined, standardized competency framework for nurses has led to considerable variability in IPC knowledge and practice, often shaped by institutional policy rather than unified standards [3]. This variability directly affects patient and healthcare worker safety.

Globally, structured, competency-based IPC education is recognized as central to patient safety and quality of care. Countries such as the United States, Canada, and Australia have established comprehensive IPC competencies aligned to professional certification and clinical roles [4-6]. The World Health Organization’s 2016 guidelines specifically identify education as a cornerstone for developing measurable IPC competencies in all healthcare professionals, both pre-service and in-service [7-9]. Recent progress in India includes the Indian Nursing Council’s revised B.Sc. nursing curriculum (2021) and the launch of an expert-level diploma in IPC by the Infection Control Academy of India and University of Hyderabad (2020) [10,11].

However, these improvements do not yet fully address foundational, nurse-specific needs, as most practicing nurses were trained prior to the introduction of such curricula, and advanced programs primarily serve a limited subset of healthcare staff. The ongoing absence of structured orientation, regular skill validation, and robust system-wide competency assessment continues to compromise safe IPC practice. There remains a critical gap in accessible, scalable, nurse-centered training that meets the diverse needs of India’s large nursing workforce [12].

To address this gap and guide pathways to universal IPC competency, this study assesses Indian nurses’ IPC knowledge, attitudes, and practices (KAP) through a nationwide survey, aligned with current global and national benchmarks [7,10,11]. The findings aim to inform practical, evidence-based nurse-centric training and certification, ultimately supporting stronger IPC implementation and better patient outcomes.

## Materials and methods

Study design and participants

A nationwide, cross-sectional, internet-based study was conducted from October to November 2023 to assess nurses' KAP related to IPC competencies. The survey link was circulated among a known group of nurses, who were encouraged to share it further with their peers through professional networks and social media platforms, thereby employing a snowball sampling approach. All nurses, regardless of age, gender, qualification (diploma, undergraduate, postgraduate), years of experience, or healthcare setting, were invited to participate. Eligibility was based on self-identification as part of the nursing workforce.

Survey platform, consent, and ethical considerations

The survey was conducted using Google Forms and developed in line with the CHERRIES (Checklist for Reporting Results of Internet E-Surveys) guidelines to ensure methodological transparency [13]. Before beginning the survey, participants were informed about the study’s purpose, voluntary nature, data confidentiality, and the principal investigator’s contact details. All questions were marked as mandatory to ensure completeness, and the Google Forms setting to limit one response per user was enabled to prevent duplicate submissions. The order of questions and response options was fixed and presented in the same sequence to all participants; no randomization or adaptive questioning was applied. Responses were anonymized, and data were stored securely with access restricted to the research team.

The study protocol was reviewed and approved by the Institutional Ethics Committee, University of Hyderabad (DHR:EC/NEW/INST/2023/3825).

Instrument development and pilot testing

A structured, self-administered questionnaire was developed based on guidelines from the World Health Organization (WHO) and Centers for Disease Control and Prevention (CDC) [14,15]. 

The questionnaire consisted of four sections: Section 1 comprises 21 knowledge items that cover IPC principles; Section 2 comprises 16 attitude items that assess perceptions and beliefs regarding IPC; Section 3 contains 23 practice items designed to evaluate the frequency and accuracy of IPC behaviors; Section 4 collects demographic and professional details, including information on prior IPC training.

The survey was divided into four screens, with each major section (knowledge, attitude, practice, and demographics/training) presented on a separate page to facilitate user navigation and minimize fatigue. Participants could navigate back and forth between sections using the "Next" and "Back" buttons to review and modify their responses prior to submission. Prior to final deployment, the survey was pilot tested, and feedback was used to revise item phrasing and response clarity.

Participant experience and feedback

Upon survey submission, participants received immediate, automated feedback on the knowledge section, indicating correct answers and brief explanations to promote self-directed learning.

Sample size estimation

Using the formula for estimating a population proportion ($n = Z&sup2;p(1-p)/E&sup2;)$, with Z = 1.96 (95% confidence), E = 0.05, and p = 0.60, the minimum required sample size was calculated as 369 [16,17]. A total of 547 complete responses were received, exceeding the required sample and enhancing the precision of the findings.

Statistical analysis

Knowledge and practice items were scored dichotomously (1 = correct, 0 = incorrect). Attitude items used a five-point Likert scale, with negatively worded items reverse-coded and responses dichotomized for analysis (1 = desirable, 0 = neutral/undesirable). The maximum possible composite score was 60, and scores were categorized as low (<50%), average (50-79%), or good (≥80%) following Fashafsheh et al [18]. Data was analyzed using SPSS version 22.0 (IBM Corp., Armonk, NY). Descriptive statistics (frequencies, percentages, means, and standard deviations) were used for demographic characteristics and KAP scores. As the data were not normally distributed, nonparametric tests were applied. The chi-square test was used to explore associations between KAP scores and demographic variables. Variables with significant associations were included in an ordinal logistic regression model to identify contributing factors. A p-value of <0.05 was considered statistically significant.

## Results

A total of 547 nursing professionals participated in the study. Of these, 445 (81.4%) were female, and 372 (68.0%) were aged between 20 and 30 years. Regarding qualifications, 247 (45.2%) held a diploma and 201 (36.7%) held an undergraduate degree. In terms of the workplace, the largest proportion were employed in corporate hospitals (n=396; 72.4%). With respect to experience, 172 (31.4%) had 1-3 years of post-graduation experience (Table 1).

**Table 1:** Demographic Characteristics of the Participants (n = 547) Values are presented as n (%), where n represents the number of participants and % indicates the corresponding percentage.

Variables	n(%)
Age	
20-30 years	372(68)
31-40 years	118(21.6)
41-50 years	47(8.6)
> 50 years	10(1.8)
Sex	
Female	445(81.4)
Male	98(17.9)
Chose not to answer	4(0.7)
Qualification	
Diploma	247(45.2)
Postgraduate	93(17)
Student	6(1.1)
Undergraduate	201(36.7)
Healthcare sector in which working	
Charitable/non-government organization	17(3.1)
Corporate hospital	396(72.4)
Fieldwork/community health	3(0.5)
Government/Public health facility	56(10.2)
Nursing home	8(1.5)
Private clinic/practitioner	67(12.2)
Years of experience after graduation	
1-3 yr	172(31.4)
5-10 yr	87(15.9)
< 1 yr	99(18.1)
>10 yr	97(17.7)
>3-5 yr	92(16.8)

**Figure 1:**
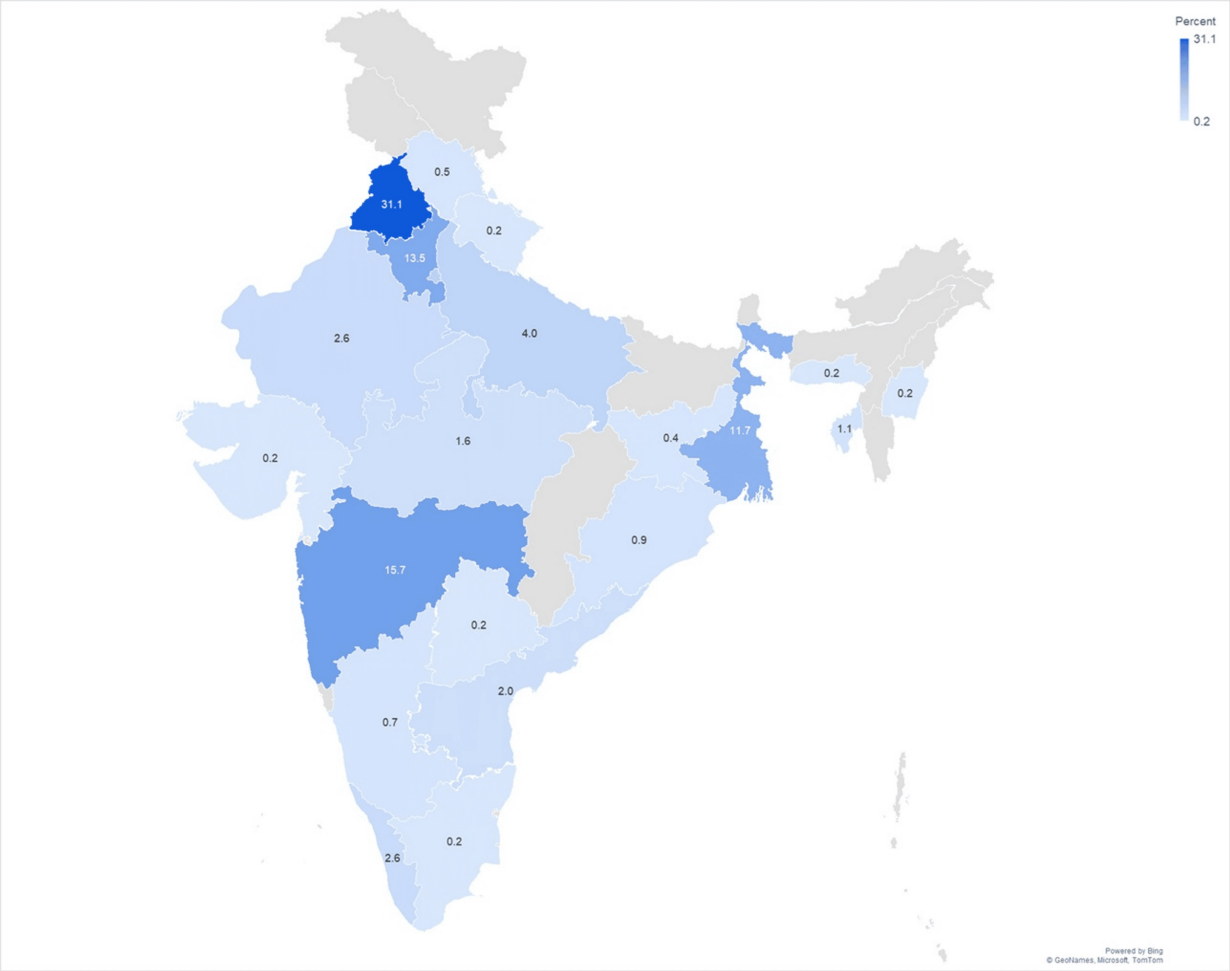
Statewise Participant Mapping Values are presented as n (%), where n represents the number of participants and % indicates the corresponding percentage.

Participants represented 22 states across India, with the highest participation from Punjab (31.1%), Maharashtra (15.7%), and Haryana (13.5%). The state-wise distribution is illustrated in Figure 1.

**Figure 2:**
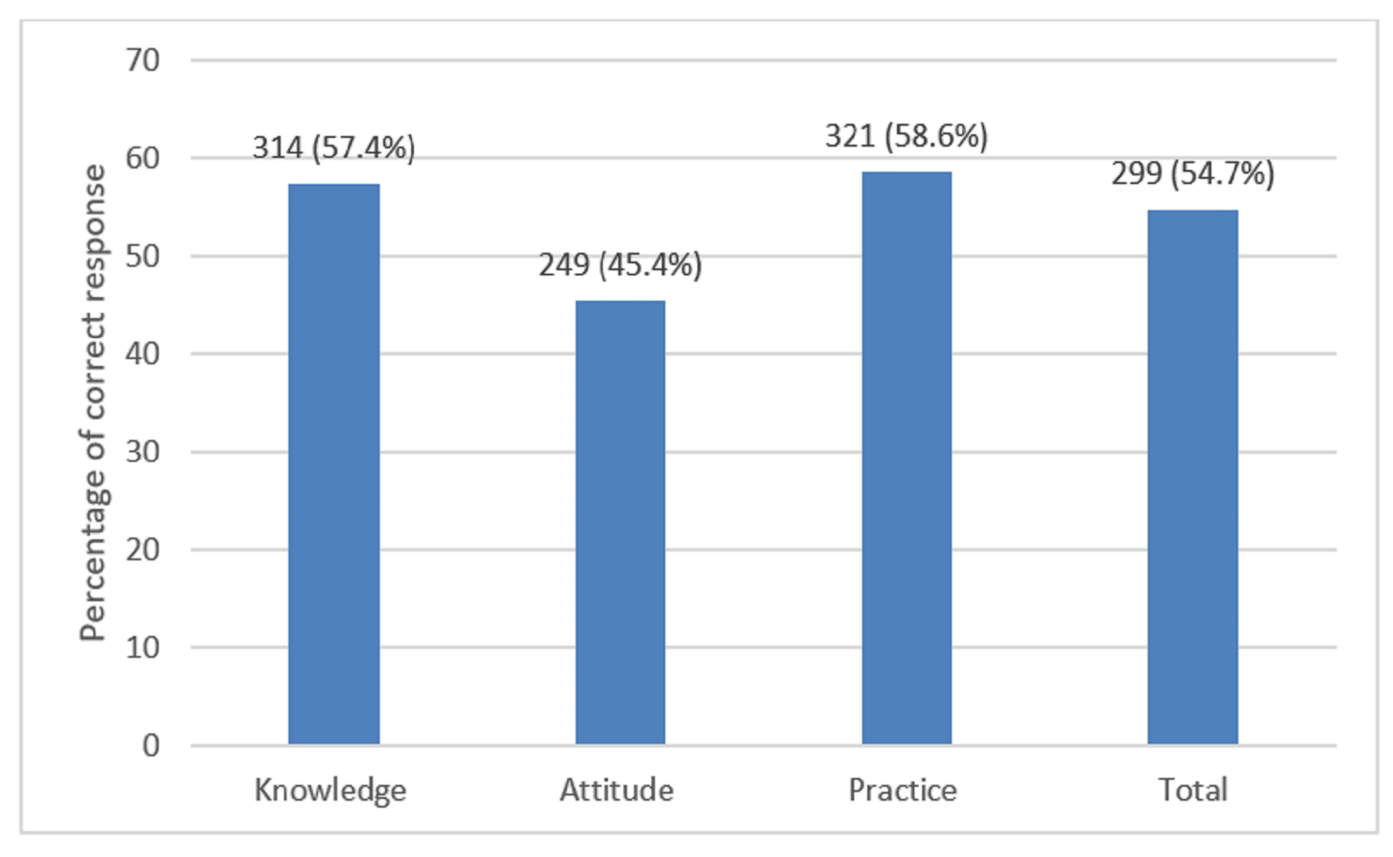
Average Scores in KAP Across All IPC Competencies (n=547) Values are presented as n (%), where n represents the number of participants and % indicates the corresponding percentage. KAP: knowledge, attitudes, and practices; IPC: infection prevention and control

The overall mean KAP score was 33.0 out of 60 (range: 18-50; SD: 5.39), corresponding to an overall correct response rate of 299 (54.7%). When analyzed by domain, the mean knowledge score was 12.1 out of 21 (range: 2-19; SD: 2.57), with a correct response rate of 314 (57.4%). The mean practice score was 13.5 out of 23 (range: 5-21; SD: 2.80), yielding a correct response rate of 321 (58.6%). Additionally, nurses’ knowledge and practice were above the overall average. By contrast, the mean attitude score was 7.2 out of 16 (range: 1-14; SD: 2.29), reflecting a less accurate response rate of 249 (45.5%), which contributed to the reduced overall performance. The distribution of correct response rates across the KAP domains is presented in Figure 2.

**Figure 3:**
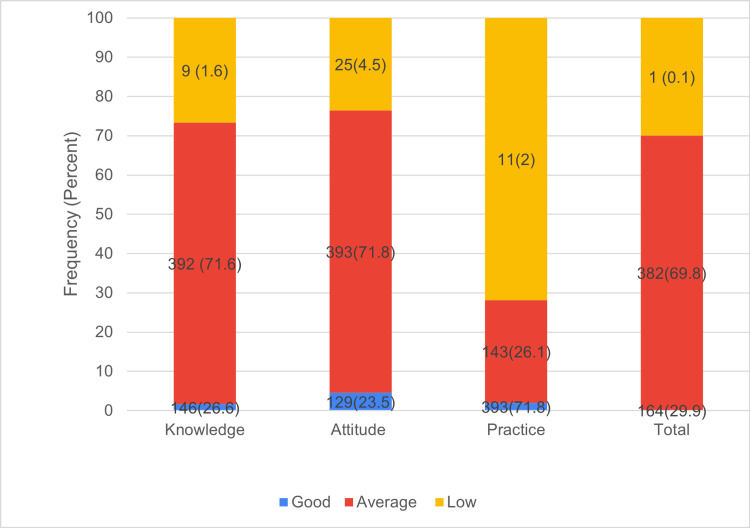
KAP Percentage Scores Categorized as Good, Average, and Low Good (≥80%), average (50%–79%), low (<50%); values are expressed as n (%), where n represents the number of participants and % indicates the percentage of the total. KAP: knowledge, attitudes, and practices

The distribution of KAP scores categorized as good (≥80%), average (50-79%), or low (<50%) showed that most participants (71%) demonstrated average levels of knowledge and attitude, as illustrated in Figure 3. In contrast, practice scores revealed a concerning gap: although the mean practice score was 58.6% within the average category but nearly three-quarters of participants (71.8%) were classified as having low practice levels, highlighting a disconnect between knowledge/attitude and actual IPC practices (Figure 3).

The distribution of questions and average positive response rates for KAP across assessed IPC competencies revealed a mixed pattern with marked variability in performance across domains and competencies, as shown in Table 2.

**Table 2:** Distribution of Questions and Percentage Scores for Correct Responses in KAP Across Assessed Competencies Values are presented as n (%), where n represents the number of participants and % indicates the corresponding percentage. KAP: knowledge, attitudes, and practices; HAI: healthcare-associated infection

Competency	K (items=21)	n (%)	A (items=16)	n (%)	P (items=23)	n (%)
Chain of transmission	4	456 (83.41)	-	-	-	-
Standard precaution applications	1	379 (69.29)	-	-	-	-
Infection prevention in specific populations	4	356 (65.17)	-	-	-	-
Antimicrobial stewardship	3	276 (50.52)	1	218(31.85)	-	-
Hand hygiene	-	-	1	335 (61.2)	-	-
Personal protective equipment	-	-	5	247(45.08)	2	215 (39.30)
Biomedical waste	1	182 (33.27)	-	-	-	-
Transmission-based precautions	1	229 (41.86)	-	-	1	99(18.10)
Occupational health and safety	1	253 (46.25)	1	318 (58.14)	2	298(54.48)
Prevention of device associated infections	1	258 (47.17)	1	375 (68.56)	9	376 (68.89)
Injection safety	-	-	-	-	7	378 (69.16)
Medical device reprocessing	1	88 (16.09)	4	234 (42.78)	-	-
Environmental control	-	-	3	192 (35.04)	-	-
Outbreak investigation and response	1	209 (38.21)	-	-	1	391 (71.48)
Surveillance of HAI	2	348 (63.71)	-	-	-	-
Risk assessment and quality improvement	1	222 (40.58)	-	-	1	76 (13.89)

Knowledge was highest for the competencies on chain of transmission (83.41%), followed by standard precautions (69.29%) and prevention of HAI in special situations (65.17%). Conversely, lower knowledge scores were observed in areas such as medical device reprocessing (16.09%) and environmental cleaning (16.45%).

The most favorable attitude was observed for prevention of device-associated infections, with 68% of nurses either agreeing or strongly agreeing. A high proportion of respondents agreed or strongly agreed with the importance of standard precautions for hand hygiene (62%) and occupational safety (58%). In contrast, standard precautions related to personal protective equipment (PPE) showed mixed responses, with 42% expressing disagreement or strong disagreement. Medical device reprocessing and environmental control also reflected moderate attitudes, with a notable proportion remaining neutral (28% and 24%, respectively). The domain of antimicrobial stewardship showed balanced responses, though a significant portion (31%) remained neutral.

Practice domain competencies varied considerably. Average adherence was reported for injection safety (69.16%) and prevention of device-associated infections (68.89%). However, critical IPC practices such as hand hygiene (37.66%), biomedical waste management (33.27%), personal protective equipment use (31.92%), and transmission-based precautions (18.10%) demonstrated suboptimal compliance. In Risk assessment and quality improvement, only 13.89% of nurses demonstrated practice-level competency.

The detailed question-wise correct response for KAP is presented in Table 3.

**Table 3:** Intent of all KAP Questions Along With Correct/Preferred Responses Values are presented as n (%), where n represents the number of participants and % indicates the corresponding percentage. KAP: knowledge, attitudes, and practices; PPE: personal protective equipment; HCW: healthcare worker; MRSA: methicillin-resistant* Staphylococcus aureus*; PEP: post-exposure prophylaxis; HAI: healthcare-associated infections

Competency	Knowledge Question Intent	Correct (n, %)	Attitude Question Intent	Correct (n, %)	Practice Question Intent	Correct (n, %)
Chain of transmission	To assess knowledge of airborne/respiratory transmission routes.	463 (84.64)	-	-	-	-
	To evaluate the understanding of vector-borne transmission via mosquitoes.	510 (93.24)	-	-	-	-
	To examine awareness of fecal-oral transmission through contaminated food/water.	360 (65.81)	-	-	-	-
	To determine knowledge of bloodborne transmission through unsafe injections or blood contact.	492 (89.95)	-	-	-	-
Standard precaution understanding	To understand the application of standard precautions in patient care	379 (69.29)	-	-	-	-
PPE	-	-	To assess attitude towards proper disposal and handling of face masks.	335 (61.24%)	To assess compliance with PPE change practices during multiple procedures on a single patient	98 (17.92%)
	-	-	To evaluate attitude towards appropriate glove use for low-risk procedures	273 (49.91%)	To evaluate adherence to the recommended sequence for safe PPE removal	332 (60.69%)
	-	-	To assess acceptance of enhanced PPE practices for high-risk exposures	240 (43.88%)	-	-
	-	-	To assess attitudes towards aseptic techniques during parenteral nutrition	161 (29.43%)	-	-
	-	-	To assess attitudes toward glove hygiene between patient contacts.	315 (57.59%)	-	-
Hand hygiene	-	-	To assess attitudes towards consistent hand hygiene during intra-patient care transitions.	244 (44.61%)	-	-
Biomedical waste	To assess awareness of biomedical waste categories that do not require on-site pre-treatment before disposal	182 (33.27)	-	-	-	-
Transmission-based precautions	To assess understanding of isolation precautions in meningococcal meningitis care	229 (41.86)	-	-	To assess compliance with contact precautions in the management of MRSA cases	99 (18.10%)
Occupational safety	To assess awareness of fitness-to-work criteria for occupational safety post-illness	253 (46.25)	To assess attitudes towards work restrictions for infected healthcare workers to prevent transmission	318 (58.14%)	To assess practice related to initiating appropriate PEP in unvaccinated HCWs after Hepatitis B exposure	298 (54.48%)
	-	-	-	-	To evaluate practical response and adherence to protocol following a needle-stick injury	451 (82.45%)
Prevention of device-associated infections	To assess awareness of correct indications for urinary catheter use.	258 (47.17)	To assess attitude towards safe enteral feeding practices through proper labeling	375 (68.56%)	To assess practice compliance in avoiding guidewire exchanges during suspected infections	182 (33.27%)
	-	-	-	-	To evaluate adherence to safe sampling practices	190 (34.73%)
	-	-	-	-	To assess practice in avoiding unnecessary use of antimicrobial flushes.	212 (38.76%)
	-	-	-	-	To evaluate timely removal of central lines to prevent infections.	360 (65.81%)
	-	-	-	-	To assess compliance with evidence-based skin antisepsis before catheter insertion.	413 (75.50%)
	-	-	-	-	To evaluate adherence to antiseptic practices before catheter access	468 (85.56%)
	-	-	-	-	To assess implementation of full sterile precautions during invasive line placement	469 (85.74%)
	-	-	-	-	To evaluate consistency in monitoring catheter sites for early signs of infection.	496 (90.68%)
	-	-	-	-	To assess the application of clinical scoring systems in vascular access care	220 (40.22%)
Infection prevention – specific situations	To assess awareness of screening protocols for bloodborne viruses in dialysis settings.	459 (83.91)	-	-	-	-
	To evaluate understanding of the need for dedicated machines for HBV-infected patients.	469 (85.74)	-	-	-	-
	To assess knowledge of safe staffing practices in relation to HBV-positive patients.	231 (42.23)	-	-	-	-
	To evaluate awareness of correct procedures for cleaning and disinfecting dialysis machines	267 (48.81)	-	-	-	-
Antimicrobial stewardship	To assess the knowledge of optimal timing for collecting cultures	339 (61.97)	To assess attitude towards the importance of sterilization and disinfection over reliance on prophylactic antibiotics	218 (39.85%)	-	-
	To evaluate awareness of key microbiological results requiring urgent action.	243 (44.42)	-	-	-	-
	To evaluate awareness of key microbiological results requiring urgent action.	247 (45.16)	-	-	-	-
Outbreak investigation and response	To assess the ability to identify a common source during an outbreak investigation.	338 (61.79%)			To assess adherence to recommended steps in managing a suspected outbreak of vomiting and diarrhea	391 (71.48%)
Medical device reprocessing	To assess understanding of the purpose of Type 1 chemical indicators in device reprocessing	88 (16.09)	To assess attitudes towards correct placement of sterilization indicators	188 (34.37%)	-	-
	-	-	To evaluate attitudes towards proper validation of sterilizer performance	209 (38.21%)	-	-
	-	-	To assess attitude towards appropriate use of flash sterilization	223 (40.77%)	-	-
	-	-	To evaluate attitudes towards following correct disinfection protocols	316 (57.77%)	-	-
Surveillance of HAI	To assess the knowledge of criteria for identifying CAUTI cases	372 (68.01)	-	-	-	-
	To evaluate understanding methods used to calculate HAI rates	325 (59.41)	-	-	-	-
Risk assessment and quality improvement	To assess awareness of risk assessment tools used during hospital renovation activities.	222 (40.58)	-	-	To assess the application of quality improvement tools for analyzing HAI outbreaks	76 (13.89%)
Environmental control	-	-	To assess attitude towards evidence-based use of environmental surveillance	217 (39.67%)	-	-
	-	-	To evaluate attitude towards appropriate methods for air disinfection	153 (27.97%)	-	-
	-	-	To assess attitude towards prioritizing effective surface disinfection practices over fogging	205 (37.48%)	-	-
Injection safety	-	-	-	-	To assess adherence to safe storage practices for multi-dose vials	166 (30.35%)
	-	-	-	-	To evaluate compliance with discarding vials when contamination is suspected	258 (47.17%)
	-	-	-	-	To assess practice of proper labeling for safe tracking and use of vials	392 (71.66%)
	-	-	-	-	To evaluate adherence to recommended timelines for vial disposal	405 (74.04%)
	-	-	-	-	To assess practice of maintaining clean and organized storage of vials	447 (81.72%)
	-	-	-	-	To evaluate compliance with aseptic technique when accessing multi-dose vials	480 (87.75%)
	-	-	-	-	To assess practice of documenting vial opening to ensure safe usage duration	500 (91.41%)

The distribution of participants’ preferences regarding training duration, certification type, mode of delivery, training format, and learning methods showed a clear inclination toward short-term intensive training (48.8%), certificate-level courses (47.2%), and practical (88.5%) as well as interactive learning approaches (84.8%), as presented in Table 4.

**Table 4:** Participants' Preferences Regarding Training Delivery Values are presented as n (%), where n represents the number of participants and % indicates the corresponding percentage.

Training preferences	n (%)
What is your preferred duration for training in infection prevention and control?	
Longer (3 months or more)	119(21.8)
Longer, comprehensive courses (1-2 weeks)	115(21)
Multiple days (2-5 days) of intensive training	267(48.8)
Others	46(8.4)
Would you prefer a training course that offers a formal document upon completion?	
Advanced Certificate in Infection Control Nursing (6 months)	148(27.1)
Certificate in Infection Control Nursing (3 months)	258(47.2)
Diploma in Infection Control Nursing (12 months)	72(13.2)
Fellowship in Infection Control Nursing	69(12.6)
Which mode of training delivery do you prefer?	
Distance learning (online, self-paced)	208(38)
Full-time (immersive, daily training)	149(27.2)
Other	28(5.1)
Part-time (few days a week or weekends)	162(29.6)
Which type of training do you prefer?	
Can’t say	94(17.2)
Hybrid	54(9.9)
Asynchronous training (self-paced modules)	171(31.3)
Synchronous training (real-time, live sessions)	228(41.7)
Would you like the training to include practical, hands-on components, such as simulations or skills labs?	
No	63(11.5)
Yes	484(88.5)
Are you open to collaborative group training or team-based learning as part of your training?	
No	83(15.2)
Yes	464(84.8)

Age, the healthcare sector of employment, and years of experience after graduation were significantly associated with both knowledge and practice levels among nurses. Additionally, educational qualifications were significantly associated with practice. No significant associations were found with attitude scores, so it was not analyzed further by logistic regression (Table 5).

**Table 5:** Association of Demographic Variables With IPC KAP Levels Chi-square (χ^2^) test was used to study the association between KAP scores and demographic variables. P-value < 0.05 was considered statistically significant. * denotes statistically significant values in the table. Values are presented as n (%), where n represents the number of participants and % indicates the corresponding percentage IPC: infection prevention and control; KAP: knowledge, attitudes, and practices

Variables	Knowledge			χ^2^, p-value	Attitude			χ^2^ , p-value	Practice			χ^2^, p-value
	Low (n=146)	Average (n=392)	Good (n=9)		Low (n=297)	Average (n=239)	Good (n=11)		Low (n=129)	Average (n=393)	Good (n=25)	
Age												
20-30 years	108(73.97%)	262(66.84%)	2(22.22%)	15.33, 0.018*	204(68.69%)	159(66.53 %)	9(81.82%)	4.44, 0.61	94(72.87%)	270(68.7%)	8(32%)	35.33, <0.001*
31-40 years	26(17.81%)	87(22.19%)	5(55.56%)		63(21.21%)	53(22.18%)	2(18.18%)		29(22.48%)	82(20.87%)	7(28%)	
41-50 years	9(6.16%)	37(9.44%)	1(11.11%)		27(9.09%)	20(8.37%)	0(0%)		5(3.88%)	35(8.91%)	7(28%)	
> 50 years	3(2.05%)	6(1.53%)	1(11.11%)		3(1.01%)	7(2.93%)	0(0%)		1(0.78%)	6(1.53%)	3(12%)	
Sex												
Female	119(81.51%)	323(82.40%)	7(77.78%)	0.17, 0.91	246(82.83%)	194(81.59%)	8(72.73%)	0.81, 0.66	105(81.4%)	321(81.68%)	23(92%)	1.76, 0.45
Male	27(18.49%)	69(17.6%)	2(22.22%)		51(17.17%)	44(18.41%)	3(27.27%)		24(18.6%)	72(18.32%)	2(8%)	
Qualification												
Diploma	69(47.26%)	173(44.13%)	5(55.56%)	10.45, 0.120	133(44.78%)	107(44.77%)	7(63.64%)	10.96, 0.090	61(47.29%)	174(44.27%)	12(48%)	17,89, 0.007*
Postgraduate	24(16.44%)	65(16.58%)	4(44.44%)		39(13.13%)	53(22.18%)	1(9.09%)		14(10.85%)	69(17.56%)	10(40%)	
Student	3(2.05%)	3(0.77%)	0(0%)		4(1.35%)	2(0.84%)	0(0%)		3(2.33%)	3(0.76%)	0(0%)	
Undergraduate	50(34.25%)	151(38.52%)	0(0%)		121(40.74%)	77(32.22%)	3(27.27%)		51(39.53%)	147(37.4%)	3(12%)	
Healthcare sector												
Charitable/non-government organization	5(3.42%)	11(2.81%)	1(11.11%)	30.05, 0.001*	5(1.68%)	12(5.02%)	0(0%)	8.08, 0.621	2(1.55%)	13(3.31%)	2(8%)	29.43, 0.001*
Corporate hospital	90(61.64%)	299(76.28%)	7(77.78%)		214(72.05%)	172(71.97%)	10(90.91%)		86(66.67%)	293(74.55%)	17(68%)	
Fieldwork/community health	2(1.37%)	1(0.26%)	0(0%)		2(0.67%)	1(0.42%)	0(0%)		0(0%)	2(0.51%)	1(4%)	
Government/public health facility	12(8.22%)	44(11.22%)	0(0%)		33(11.11%)	22(9.21%)	1(9.09%)		11(8.53%)	41(10.43%)	4(16%)	
Nursing home	5(3.42%)	3(0.77%)	0(0%)		5(1.68%)	3(1.26%)	0(0%)		6(4.65%)	2(0.51%)	0(0%)	
Private clinic/practitioner	32(21.92%)	34(8.67%)	1(11.11%)		38(12.79%)	29(12.13%)	0(0%)		24(18.6%)	42(10.69%)	1(4%)	
Years of experience after graduation												
1-3 yr	63(43.15%)	109(27.81%)	0(0%)	42.30, <0.001*	95(31.99%)	71(29.71%)	6(54.55%)	9.65, 0.290	42(32.56%)	128(32.57%)	2(8%)	48.19, <0.001*
5-10 yr	19(13.01%)	66(16.84%)	2(22.22%)		51(17.17%)	35(14.64%)	1(9.09%)		21(16.28%)	62(15.78%)	4(16%)	
< 1 yr	21(14.38%)	78(19.9%)	0(0%)		51(17.17%)	45(18.83%)	3(27.27%)		27(20.93%)	69(17.56%)	3(12%)	
>10 yr	14(9.59%)	76(19.39%)	7(77.78%)		45(15.15%)	51(21.34%)	1(9.09%)		11(8.53%)	70(17.81%)	16(64%)	
>3-5 yr	29(19.86%)	63(16.07%)	0(0%)		55(18.52%)	37(15.48%)	0(0%)		28(21.71%)	64(16.28%)	0(0%)	

Ordinal logistic regression was performed to identify factors associated with knowledge and practice. Years of professional experience and practice levels were significantly associated with knowledge among nurses, as shown in Table 5.

Nurses with less than 1 year, 5-10 years, and more than 10 years of experience had significantly higher odds of having better knowledge compared to those with 1-3 years of experience. The likelihood of better knowledge increased with increasing years of experience; nurses with more than 10 years of experience showed the strongest association. Knowledge was also strongly linked to practice. Nurses with good practice scores (80% or above) were significantly more likely to have good knowledge. In contrast, those with average practice scores (50-79%) had much lower odds of having good knowledge, and nurses with low practice scores (less than 50%) were the least likely to demonstrate good knowledge. Although age appeared significant in the unadjusted analysis - participants aged 31-40 years had higher odds of better knowledge compared to those aged 20-30 years - this association was not significant after adjustment. Educational qualification also showed no significant association with knowledge.

Educational qualifications and professional experience were significantly associated with practice levels. Nurses with postgraduate qualifications were more likely to demonstrate good practice compared to those with diploma-level education. Similarly, those with more than 10 years of experience had significantly better practice than those with one to three years of experience.

**Figure 4:**
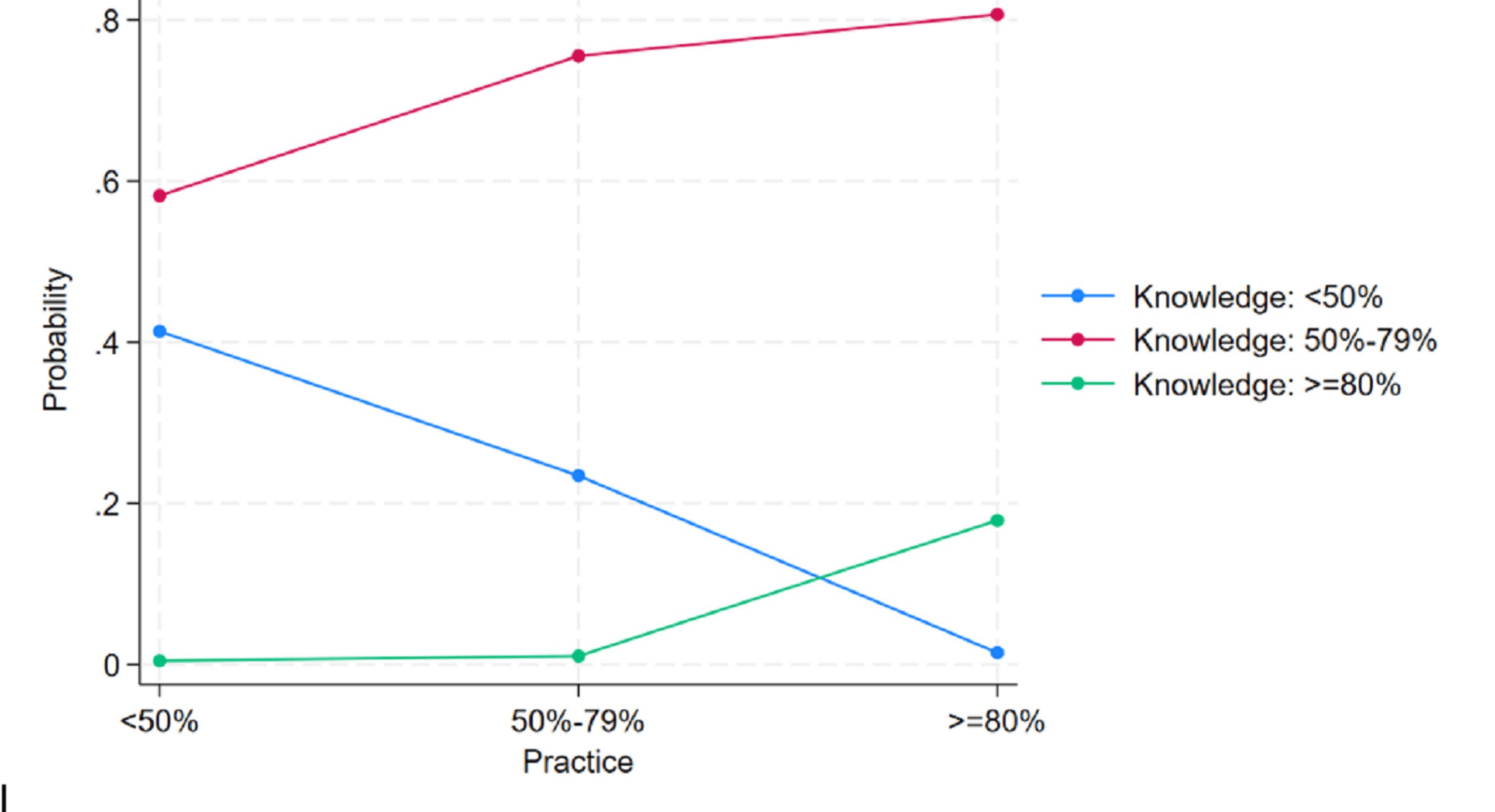
Predicted probabilities by practice level Ordinal logistic regression was used to assess the association

The predicted probabilities highlight a clear trend between knowledge and practice. Nurses with lower knowledge scores are more likely to remain in the low practice category. As knowledge levels increase, the likelihood of having better practices also rises, indicating a positive relationship between knowledge improvement and regular practice (Figure 4).

## Discussion

Identifying specific IPC competencies among nurses is vital for designing effective, competency-based education and performance monitoring systems. This nationwide study provides a comprehensive, nurse-specific assessment of KAP across diverse IPC domains in India. Unlike previous studies that were limited by geography, cadre mix, focus on only one domain, or basic assessment techniques, our study aligns with globally recognized IPC competencies and offers important insights for strengthening IPC capacity among frontline nursing staff [16,19-21].

In this study, participants showed average overall IPC competency, with just over half answering correctly across KAP domains. Notable gaps were seen in attitude and especially in practice. Although the mean practice score was 58.6%, more than 70% of nurses fell into the "low" category, indicating systemic barriers such as resource limitations, high patient load, and a lack of regular feedback that hinder effective application of IPC knowledge in daily work [20]. These findings highlight that it's not enough to focus on knowledge acquisition alone; strategies are needed that help nurses confidently apply IPC principles in routine clinical care [20]. Our findings differ from a previous study in super-specialty hospitals, where attitude scored highest (73%) and knowledge lowest (51%) [15]. This difference likely reflects our broader, more diverse sample and the use of a structured, competency-based tool, showing how institutional context strongly influences nurse performance.

It is challenging to directly compare the IPC scores from our study with other research because of major methodological differences. Most previous studies assessed IPC practices using basic yes-or-no questions such as "Does hand hygiene prevent HAIs?" and often focused on just one or two specific competencies, like hand hygiene, PPE use, antimicrobial stewardship, etc. [17,19-23]. Because these questions were relatively simple and didn’t measure how well nurses understood or performed these tasks, these studies probably overestimated nurses' competency in IPC [17,19-23]. Furthermore, a majority of these studies were conducted in high-income countries or within specific hospital types, limiting their generalizability to broader healthcare settings [19,23,24]. The high variability in reported findings across studies highlights how much factors such as institutional support, work setting, and assessment methods impact IPC results [16-19,21,25]. This makes clear the need for standardized, context-sensitive tools to assess and strengthen IPC skills among healthcare workers.

While foundational IPC concepts (e.g., chain of transmission, standard precautions) were well understood, knowledge in areas like device reprocessing, risk assessment, and antimicrobial stewardship was lower, suggesting gaps in training content or clinical exposure. Attitude deficits were most evident for outbreak investigation and antimicrobial stewardship, reflecting limited confidence but also constraints in interdisciplinary collaboration and mentorship. Key practice gaps, especially in hand hygiene, PPE use, transmission-based precautions, and equipment reprocessing, seem related to both skill deficits and systemic issues like supply shortages, large patient volume, and lack of compliance incentives.

Experience appeared more influential than age or education in IPC performance, with both newly employed and highly experienced nurses scoring better than those with 1-3 years of experience. This could be due to initial enthusiasm or cumulative clinical exposure, while mid-career nurses may be navigating role changes, thereby finding it challenging to integrate into daily routines. In contrast, formal education had only a modest effect on practice and little effect on knowledge or attitude, while workplace culture and visible role modeling showed a greater impact, supporting findings from national studies where knowledge is often linked to accreditation and work area. Overall, we found a consistent disconnect between knowledge and attitude, and a clearer link between knowledge and actual practice. This points to a need for training that goes beyond theory to include hands-on experiences, behavioral reinforcement, and workplace norms. Strategies such as simulation, mentorship, and reflective learning are likely to be more effective for sustained behavior change than didactic instruction alone [20].

These findings echo global guidance that IPC education should transition from lectures to real, experiential learning. Competency-based approaches, including simulation, peer mentoring, and routine feedback, are especially valuable for nurses in transitional career stages and in areas where practical deficits are greatest [25,26]. Embedding IPC competencies into clinical routines and linking them to structured monitoring (like audits and peer feedback) is essential for lasting improvement. Well-resourced IPC programs integrated into core healthcare operations are also crucial. As India faces new infectious threats, disaster-preparedness IPC training must be included in nursing education and ongoing professional development to support resilient systems and ready frontline staff. Although this study centers on India, the findings are highly relevant across the Global South, where similar challenges of infrastructure, training, and infection risk are very common. Addressing these gaps is not only vital for India but for the entire region.

To translate these findings into meaningful action, we propose some practical steps. Nursing schools and hospitals should include practical IPC training in all nursing courses - undergraduate, diploma, and postgraduate - to ensure every nurse gets hands-on experience. Nurses should be able to demonstrate their infection control skills at different career stages and earn certification as they progress. Training should use real-life scenarios, simulations, and peer mentoring to prepare nurses for clinical practice, not just theoretical knowledge. Hospitals can organize regular peer reviews and group sessions to encourage learning and provide feedback. Adding disaster and outbreak preparedness training will help nurses handle emergencies better. Above all, these efforts should ensure equal access to training and resources for nurses in smaller or rural hospitals, so every nurse is included.

This study's strengths include being one of the largest nurse-specific IPC assessments in India, offering detailed insights across diverse clinical settings. The design emphasized higher-risk domains for greater practical relevance. However, the study relied on self-reported data, which may have introduced bias if participants overstated their compliance. Voluntary participation may have drawn more IPC-interested nurses, limiting generalizability, and uneven domain item distribution restricted direct comparisons between areas.

## Conclusions

This study reveals substantial gaps in IPC competencies among Indian nurses, with the most significant weaknesses observed in attitude and daily practice. Our findings clearly show that knowledge alone is not enough to drive lasting behavior change; instead, consistent and confident application of IPC principles in clinical care requires competency-based, hands-on training and ongoing support. Effective solutions will demand both policy-level leadership and strong institutional mechanisms to make such training accessible and sustainable. The development of scenario-based, experiential IPC modules offers a promising pathway for translating theoretical understanding into practical skills. By embedding these strategies within routine nursing education and workplace operations, India can build a resilient healthcare workforce, strengthen infection prevention, and better prepare for emerging public health threats. Ultimately, closing these competency gaps will improve patient safety and support higher standards of care nationwide.
